# Role of Serine Proteases at the Tumor-Stroma Interface

**DOI:** 10.3389/fimmu.2022.832418

**Published:** 2022-02-11

**Authors:** Ravichandra Tagirasa, Euna Yoo

**Affiliations:** Chemical Biology Laboratory, National Cancer Institute, Frederick, MD, United States

**Keywords:** tumor-stromal interaction, serine protease, fibroblast activation protein, urokinase plasminogen activator, kallikrein, granzyme, extracellular matrix remodeling, signaling pathways

## Abstract

During tumor development, invasion and metastasis, the intimate interaction between tumor and stroma shapes the tumor microenvironment and dictates the fate of tumor cells. Stromal cells can also influence anti-tumor immunity and response to immunotherapy. Understanding the molecular mechanisms that govern this complex and dynamic interplay, thus is important for cancer diagnosis and therapy. Proteolytic enzymes that are expressed and secreted by both cancer and stromal cells play important roles in modulating tumor-stromal interaction. Among, several serine proteases such as fibroblast activation protein, urokinase-type plasminogen activator, kallikrein-related peptidases, and granzymes have attracted great attention owing to their elevated expression and dysregulated activity in the tumor microenvironment. This review highlights the role of serine proteases that are mainly derived from stromal cells in tumor progression and associated theranostic applications.

## Introduction

**Figure 1:**
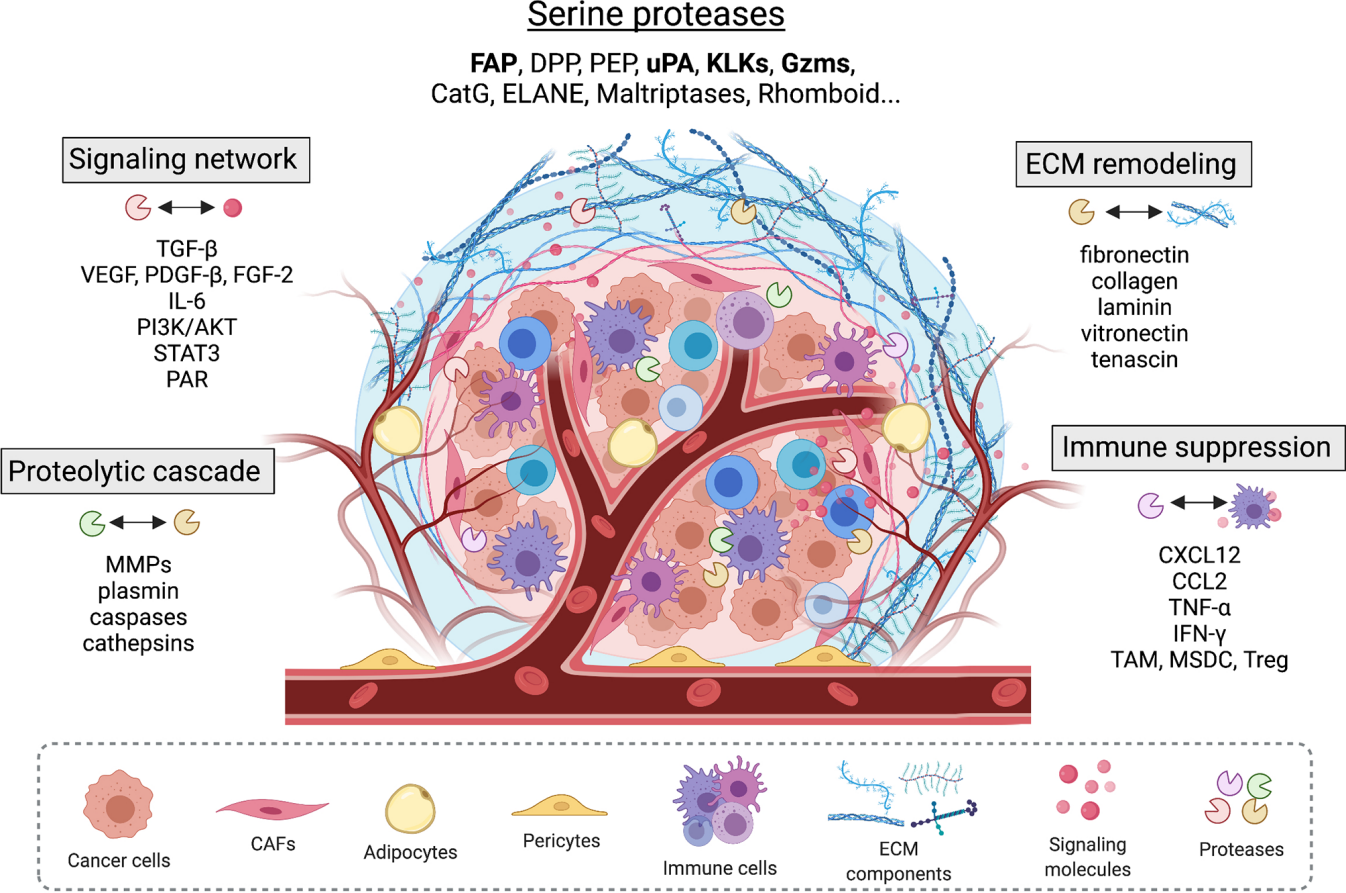
Serine proteases found in the tumor microenvironment modulate tumor-stromal interplay by interacting with other proteases, extracellular matrix proteins, and important signaling pathways that involve growth factors, cell surface receptors, cytokines and chemokines.

The tumor microenvironment (TME) is a highly complex system that is comprised of a heterogenous population of cancer cells and associated stromal cells, and the extracellular matrix (ECM). The ECM not only provides structural support in the extracellular space but also regulates multiple cellular signaling in tumor tissues (1, 2). The tumor stroma, as a critical component of the TME, actively contributes to cancer proliferation, angiogenesis, invasion and metastasis, immune evasion, and resistance to cancer therapy (3, 4). The function of stromal cells and their interaction with cancer cells within the TME are modulated by expression and secretion of various signaling molecules such as growth factors (5–7), chemokines (8–10), cytokines (11–14), and proteolytic enzymes (15–17). Although once thought to be limited to the degradation of ECM, the role of proteases in tumors is now better understood to be significantly more complicated and critical. In addition to cancer cells, stromal cells including fibroblasts, endothelial cells and infiltrating immune cells all contribute proteases in developing tumors such as matrix metalloproteinases (MMPs), cysteine cathepsins, and serine proteases. Proteases are involved in proteolytic networks, remodeling of ECM, regulation of growth factor and cytokine signaling, and modulation of inflammatory responses and immunosuppressive effects (**Figure 1**). Depending on the cellular context, activity and interaction of these proteases can have either tumor-promoting or -suppressing effects. Among, serine proteases, which make up approximately one-third of human proteases, are important class of proteases in carcinomas. Dysregulated expression and activity of several serine proteases that are derived from stromal cells have been associated with tumor development and metastasis. In this review, we aim to summarize our current understanding of functional roles that key serine proteases play in the transformed stroma and discuss their theranostic potential.

## Fibroblast Activation Protein α (FAPα, FAP or Seprase)

Cancer-associated fibroblasts (CAFs), which are perpetually activated and distinct from normal fibroblasts in their morphological and functional features, are extremely abundant in the tumor microenvironment including breast, prostate, and pancreatic carcinomas with potent tumorigenic effects (18). CAFs are highly heterogeneous, proliferative, and are resistant to apoptotic cell death. Among several surface bound markers identified in CAFs, fibroblast activation protein α (FAP) is shown to be selectively upregulated on reactive stromal fibroblasts of more than 90% of human epithelial carcinomas (19). It has been demonstrated that altered tumor microenvironment or inflammation induces FAP expression through stimulation of cytokines such as TGF-β1 and TNF-α (20, 21), chemical substances (22), or physical stimulants (23). FAP is a type II transmembrane glycoprotein that displays both dipeptidyl peptidase and endopeptidase activities, removing two amino acids from the N-terminus of the substrate as well as hydrolyzing peptide bonds of nonterminal amino acids after a proline residue. The postprolyl peptidase activity of FAP requires homodimerization of the protein and is carried out by the catalytic triad consisting of Ser, Asp, and His residues (24). Physiological substrates of FAP include gelatin, denatured type I collagen, α-antitrypsin, α2-antiplasmin, fibroblast growth factor 21, fibrillin-2, extracellular matrix protein 1, C-X-C motif chemokine 5, tumor necrosis factor related protein 6, lysyl oxidase homolog 1, and several neuropeptides (25–27). Protease-independent activity of FAP has been associated with activation of MMP2/9, phosphoinositide 3-kinases (PI3Ks), and STAT signaling pathways (28, 29).

Given its high expression on CAFs and ability to degrade major ECM proteins, the role of FAP in remodeling and patterning of ECM, which in turn affects cellular response and tumorigenesis, has been investigated. In both syngeneic transplant and endogenous mouse tumor models, genetic deletion of FAP or pharmacologic inhibition of its enzymatic activity led to excessive accumulation and disorganization of collagen and decrease in myofibroblast content and blood vessel density in tumors, thus inhibiting tumor growth (30). Utilizing an *in vivo*-like 3-dimensional matrix system, Lee et al. demonstrated that FAP overexpression on fibroblasts modifies architecture and composition of ECM through inducing tumor stromal-like parallel organization of fibronectin and collagen I fibers and modulating protein levels of tenascin C, collagen I, fibronectin and α-smooth muscle actin (31). Enhanced velocity and directionality of pancreatic cancer cells invading through FAP^+^ matrices were observed, which was effectively reversed by inhibition of FAP enzymatic activity. *In vitro*, a study showed that compared to control transfectants that do not express FAP and form slow growing tumors, cells expressing not only wild type FAP but also catalytic mutant of FAP degrade fibronectin matrices more extensively, accumulate higher levels of MMP-9, invade type I collagen gels to a significantly higher degree, and have altered pattern of tyrosine phosphorylated proteins, suggesting the functions of FAP independent of its enzymatic activity (32). FAP is found to form a protease complex with dipeptidyl peptidase IV (DPP4), which is another type II transmembrane protein with serine protease activity, at the endothelial cells of capillary-like micro-vessels within invasive breast ductal carcinoma. *In vitro* experiments showed that gelatin-binding domain of DPP4 brings this DPP4-FAP complex together with gelatin substrates at the migratory endothelial cells facilitating the local degradation of the extracellular matrix and subsequent cell migration and invasion (33). Analysis of gastric cancer (GC) patient tissue samples showed that FAP expression is positively correlated with micro-vessel density indicating the role of FAP expression in angiogenesis and metastasis (34). Many studies have demonstrated that FAP expression regulates signaling pathways that control cell cycle, proliferation, migration, and invasion (29, 35–37).

Growing evidence has also suggested that FAP^+^CAFs can promote the immunosuppressive tumor microenvironment. When FAP^+^ cells were depleted in lung or pancreatic cancers, it caused immediate growth arrest of immunogenic tumor through TNF-α- and IFN-γ-mediated mechanism (38). The study by Feig et al. showed that production of chemokine (C-X-C motif) ligand 12 (CXCL12) by FAP^+^CAFs mediates the immune suppressive activity and accounts for the failure of T cell checkpoint inhibitors in pancreatic ductal adenocarcinoma (PDA) (39). Combining with administration of α-CTLA-4 or α-PD-L1, depletion of FAP^+^ cells or inhibiting CXCR4 (CXCL12 receptor) diminished PDA growth. FAP also induces inflammatory CAFs by STAT3 activation leading to increased expression of CCL2, which promotes the tumor recruitment of myeloid-derived suppressor cells (MDSCs) and immunosuppression (40, 41). In murine models of pancreatic adenocarcinoma, inhibition of FAP proteolytic activity resulted in decreased macrophage recruitment, and genetic knockout of FAP enhanced T cell infiltration and cytotoxicity (42). However, in another study in non-small cell lung cancer (NSCLC), high density of FAP^+^CAFs was found to be associated with improved prognosis in patients with high expression of CD8 and CD3 T lymphocytes (43, 44). This result is contrary to previous finding in a small study (n= 59) that higher levels of FAP expressing stromal cells are associated with worse overall survival and increased peripheral neutrophil and lymphocyte count ratio (NLR) in NSCLC patients (45). In invasive ductal carcinoma of the breast, FAP^+^CAFs have been associated with longer survival (46), while in pancreatic adenocarcinoma and rectum they are found to be associated with worse clinical outcome (47–49). Thus, the cellular context and mechanism of FAP on the antitumor immune responses in the TME would require further investigation.

## Urokinase Plasminogen Activator (Urokinase-Type Plasminogen Activator, Urokinase, uPA)

Urokinase plasminogen activator (uPA) is a serine protease that is extracellularly localized and involved in the plasminogen activator system. It was first found in the urine (50), but later identified also in plasma, seminal fluid, and the extracellular matrix (51). It is synthesized and secreted as an inactive zymogen known as pro-uPA and different proteases such as cathepsin B and L, trypsin, kallikrein, and mast cell tryptase convert pro-uPA into an active uPA (52, 53). Binding of uPA to uPA receptor (uPAR), which is a glycosyl-phosphatidylinositol (GPI)-anchored cell membrane receptor highly expressed in most types of solid tumors such as breast, prostate, brain, and head and neck cancers, localizes the active uPA to the cell surface and converts its major substrate plasminogen into plasmin. Once activated, non-specific protease plasmin is involved in degradation of collagen IV, laminin, fibronectin, vitronectin, fibrin, and several blood clotting factors either directly or through activation of other proteases such as MMPs (54). The inhibitory proteins, plasminogen activator inhibitor-1 (PAI-1) and PAI-2, regulate the activity of uPA (55).

The role of uPA in tumor invasion and metastasis has been widely investigated. Depending on cancer type, uPA and uPAR are expressed both by cancer and stromal cells. uPA is mainly expressed by tumor-associated macrophages (TAMs) and CAFs, and on tumor endothelial cells (TECs) to lesser extent. Through interaction between uPAR and integrins as well as ECM components such as vitronectin, uPA system regulates cell adhesion and migration. uPA supports tumor cell proliferation by proteolytically activating various growth factors that include epidermal growth factor (EGF), fibroblast growth factor-2 (FGF-2) and hepatocyte growth factor/scatter factor (HGF-SF) (56). TGF-β, which is a predominant and multifunctional cytokine found in the TME, regulates the expression of uPA in several types of transformed cells (57–59). uPA-activated plasmin, in turn, activates the secreted TGF-β precursor by a proteolytic cleavage within the N-terminal region of latency-associated peptide (60–62). This loop contributes to tumor growth, cancer cell migration, epithelial to mesenchymal transition (EMT), and metastasis. uPA also mediates the effects of vascular endothelial growth factor (VEGF), a key factor in angiogenesis (63). A study showed that downregulation of uPA/uPAR inhibits angiogenesis in glioblastoma and endothelial cells by regulating tissue inhibitors of metalloproteinase-1 (TIMP-1) secretion, subsequently enhancing secretion of sVEGFR1, a known scavenger of VEGF (64). *In vitro*, uPA derived from CAFs was found to promote esophageal squamous cell carcinoma (ESCC) cell proliferation, migration, and invasion by activating PI3K/AKT and ERK signaling pathways (65). In multiple myeloma, increased expression and activity of both uPA and uPAR on CAF cells with higher proliferative rate and invasion potential were observed, suggesting the potential role of uPA/uPAR system in promoting metastasis of malignant plasma cells (66). uPA/uPAR system has been implicated in suppression of apoptotic cell death. RNAi-mediated downregulation of uPA and uPAR led to dephosphorylation of focal adhesion kinase (FAK), p38 MAPK, janus kinase (JNK) and ERK1/2 which in turn activates caspase-8, cytochrome c release, PARP cleavage, and subsequent apoptosis of human glioma cells (67). The uPA/uPAR system also plays a critical role in macrophage infiltration (68). TAMs, one of most abundant types of tumor-infiltrating immune cells found in the TME, exhibit important functions in tumor growth, metastasis, angiogenesis, and immune regulation. For example, by producing cytokines, chemokines, growth factors, and triggering the inhibitory immune checkpoint proteins release in T cells, TAMs promote immunosuppression (69). In addition, expression of uPAR and PAI-1 in TAMs has been correlated with vessel remodeling and node status and tumor grade, indicating that TAMs have an important role in the expression and regulation of uPA system for establishing the vascular network in tumors (70).

## Kallikrein Related Peptidases (KLKs)

Kallikrein or kallikrein related peptidases are a family of secreted serine proteases that play important roles in ECM remodeling, angiogenesis, skin homeostasis, innate immunity, male reproduction, tooth enamel formation, and neural development. In humans, there are 15 secreted KLKs (KLK1-15). KLK1-2, KLK4-6, KLK8, and KLK10-15 have trypsin-like and KLK3, KLK7, and KLK9 have chymotrypsin-like activities. Aberrant expression of KLKs has been associated with a variety of malignancies, thus the potential of KLKs as cancer markers has been suggested for several members of this protease family. In particular, because of the restricted expression in prostate, KLK3, also known as a prostate-specific antigen (PSA), has been widely employed as a clinical biomarker for prostate cancer. Laser cell microdissection analysis and immunochemistry in human breast cancer surrounding stromal cells showed significant upregulation of KLK4 but downregulation of all other KLKs (71). Immunohistochemistry analysis of tissue sections of ovarian and melanoma patients found the overexpression of KLK6 in tumor associated stromal cells and keratinocytes (72, 73). In pancreatic ductal adenocarcinoma, immunostaining analysis of epithelial tumor cells and the surrounding stroma and immune cells showed that high KLK6 protein levels in the tumor and immune cells are significantly associated with shorter survival compared to low protein levels (74). mRNA analysis of colorectal cancer (CRC) tissue samples from 136 patients showed upregulated KLK10 expression (75). Significantly increased expression of KLK10 has been also found in ovarian cancer tissues (76).

During the tumor progression, KLKs from cancer and stromal cells are released into the TME, where they can exert their proteolytic activity, mainly activating signaling networks and modulating the expression of genes and proteins important to tumor growth and invasion. For example, KLKs activate EGFR and protease-activated receptor (PAR), resulting in the stimulation of ERK1/2 signaling and enhanced subsequent cell proliferation (77–79). Analyzing the secretome of endothelial cells, a study showed that KLK12 can catalyze the release of PDGF-β from ECM components and cell surface. The released PDGF-β then mediates secretion of VEGF and angiogenesis. In addition, KLK12 has been shown to cleave the ECM proteins fibronectin and tenascin (80). KLK12-mediated remodeling of fibronectin matrix led to an increase in endothelial cell migration which was inhibited by a polyclonal antibody directed against the KLK12 cleavage site (81). An *in vitro* study showed that KLK14 recognizes and hydrolyzes pro-MMPs to active MMPs, especially the membrane-type MMPs (82). Combined expression of KLK4-7 in ovarian cancer has been associated with increased level of TGF-β1, neural cell adhesion molecule L1 (L1CAM), and other tumor-associated factors such as keratin 19 and moesin, indicating the impact of KLK proteases on the secreted proteomes shaping tumor microenvironment (83, 84). KLK4, highly expressed in prostate cancer, promotes CAF differentiation. Through PAR1 activation, KLK4 regulates FGF-1, tasgelin, and lysyl oxidase, and several soluble factors in the prostate stromal cell secretome (85). KLK4 can also stimulate uPA/uPAR system and activate MMPs, leading to the ECM degradation. Overexpression of KLK7 in melanoma cells was shown to induce a decrease in cell proliferation and colony formation but an increase in cell motility and invasion possibly through modulating cell adhesion molecules such as E-cadherin and MCAM/CD146 (86). Kallikreins have been also associated with infiltration of immune cells (87). Although many more studies are needed to better define the pathophysiological functions of each KLK in cancer, emerging evidence suggest that KLKs have significant effect on the tumor-microenvironment interaction and targeting specific KLK may provide therapeutic benefits in cancer therapy.

## Granzymes (Gzms)

Granzymes are cell death-inducing serine proteases primarily known for their role in eliminating infected and transformed cells through cytotoxic T cells and natural killer cells. Among five granzymes identified in humans (A, B, H, K, and M), GzmA and GzmB have been most widely studied. The expression of granzymes is regulated by many factors including receptor engagement and stimulation with cytokines. Granzymes are expressed as an inactive pro-enzyme that requires cleavage of N-terminal dipeptide inside secretory granules by cathepsin C (88). As granzyme is optimally active at neutral pH, granzymes stored in acidic granules are quiescent and become enzymatically active following release from the granules into the cytoplasm. In addition, the presence of protein inhibitors such as serpins regulates the proteolytic activity of granzymes (89).

Infiltrating immune cells within the TME significantly contribute to tumor suppression (immune surveillance) or tumor promotion (inflammation and angiogenesis) either directly or through the interplay with other stromal components. In regard to anti-tumor immunity, the intracellular role of granzymes is well characterized and appreciated. Upon activation, the pore-forming protein perforin helps deliver GzmB into the cytosol of target cells, where it induces apoptosis by caspase-dependent and -independent mechanisms. Activation of gasdermin B (GSDMB) by GzmA (90) or GSDME by GzmB (and indirectly by caspase-3) initiates pyroptotic cell death in tumors (91). Mounting evidence suggests that granzymes are also active players in immune regulatory cells and tumor cells. Regulatory T cells (Tregs), MDSCs, dendritic cells (DCs), mast cells, and Bregs are found to express granzymes. If not controlled, granzymes can cause self-inflicted damage of expressing cells. It has been suggested that GzmB is involved in Treg-mediated suppression and elimination of activated CTL/NK cells and antigen-presenting cells, indicating Treg cells utilize GzmB to suppress immune responses and tumor clearance, thus depending on the relative abundance of these cells in the tumor, GzmB can have either detrimental or protective function in antitumor immunity (92, 93).

In addition, extracellular (perforin-independent) functions of granzymes are emerging. Although it remains unclear what stimuli and signaling pathways regulate granzyme release, studies have long shown that patients with infectious diseases and certain proinflammatory conditions have elevated levels of extracellular GzmA/B. GzmB is also found to be constitutively released from CTL/NK cells *in vivo* (94). Once released, they can mediate the cleavage of extracellular matrix, cell surface receptors, cytokines, and act as proinflammatory proteases. The ECM substrates of GzmB include fibronectin, vitronectin, aggrecan, laminin, and decorin. D’Eliseo et al. showed that GzmB expressed in bladder cancer cell lines and urothelial carcinoma tissues is active in catalyzing vitronectin cleavage, and inhibition of GzmB activity suppresses bladder cancer cell invasion (95). A recent study also demonstrated that active GzmB released from migrating CTLs contribute to extravasation and homing of CTLs *via* basement membrane cleavage and remodeling. GzmB-null CTLs exhibited impaired homing and decreased transmigration through the vessel wall in mouse models of viral infection and inflammation. *In vitro* migration assays using Matrigel or Madin-Darby canine kidney (MDCK) cell basement membrane showed that active GzmB released from migrating CTLs enabled chemokine-driven movement and cleavage of basement membrane components (96). GzmM expressed in carcinomas has been implicated in promoting tumor growth, metastasis, and EMT dependent on STAT3 signaling (97). GzmM expression is positively related to IL-6 and VEGF release from cancer cells. Recently, extracellular GzmA was found to be engaged in gut inflammation in colorectal cancer by inducing NF-kB-dependent IL-6 production in macrophages leading to STAT3 activation (98). In mouse models, GzmA knockout or inhibition reduced inflammation and CRC development, suggesting that development of effective GzmA inhibitors could offer therapeutic benefits treating gut inflammation and CRC.

## Targeting Serine Proteases in Tumor Stroma

With respect to the clinical applications in cancer diagnosis and therapy, a number of tools that either detect the protein expression or harness and leverage the specific protease activity have been developed (**Table 1**).

**Table 1:** Theranostic targeting of serine proteases.

Target	Agent	Function	Cancer model tested	Ref.
FAP	Sibrotuzumab	humanized monoclonal antibody (mAbF19)	metastatic colorectal cancer	(99)
	FAP5-DM1	monoclonal antibody	epithelial cancer xenograft model	(100)
	OS4 TTS	bispecific antibody	fibrosarcoma cell line	(101)
	PT-100 (Val-BoroPro)	FAP and DPP inhibition	metastatic colon cancer	(102)
	PT-630 (Glu-BoroPro)	FAP and DPP4 inhibition	metastatic colorectal cancer	(30)
	M83	FAP and PREP inhibition	lung and colon cancer xenograft	(103)
	UAMC-1110 (SP-13786)	FAP and PREP inhibition	pancreatic adenocarcinoma mouse model	(42)
	FTPD	FAP-targeting prodrug of doxorubicin	breast cancer mouse model	(104)
	ASGPAGP-A12ADT	FAP-targeting prodrug of thapsigargin	breast cancer xenograft	(105)
	^131^I-mAbF19	SPECT imaging	breast adenocarcinoma and prostate cancer xenograft	(106)
	^99m^Tc-FAPI-34	SPECT imaging	metastasized ovarian and pancreatic cancer	(107)
	^68^Ga-FAPI-04, FAPI-74	PET/CT imaging	28 different cancers, gastric and lung cancer	(108–110)
	^177^Lu-FAP-2286	peptide-targeted radionuclide therapy	pancreatic, breast, rectal and ovarian cancer	(111)
	ANP_FAP_	NIR fluorescent imaging	glioblastoma xenograft	(112)
	HCFP	NIR fluorescent imaging	breast cancer mouse model	(113)
uPA	ATN-291	monoclonal antibody	prostate cancer	(114)
	Amiloride, HMA	uPA inhibition	metastatic lung and pancreatic cancer xenograft, cervical cancer	(115, 116)
	B-428, B-623	uPA inhibition	fibrosarcoma, prostate and breast cancer mouse models	(117, 118)
	WX-671, WX-UK1	uPA inhibition	breast and cervical cancer, head and neck squamous cell carcinoma	(119, 120)
	UK122	uPA inhibition	pancreatic cancer	(121)
	AF680-U33 IgG	NIR fluorescent imaging	prostate cancer xenograft	(122)
	^111^In-U33 IgG	SPECT/CT imaging	prostate cancer xenograft	(122)
	^89^Zr-Df-ATN-291	PET imaging	glioblastoma xenograft	(123)
	P-Dex	NIR fluorescent and photoacoustic imaging	breast cancer xenograft	(124)
	PB1	Antibody prodrugs	lung cancer xenograft	(125)
KLKs	FE999024	KLK1 inhibition	breast cancer cell line	(126)
	MDPK67b	KLK2 inhibition	prostate cancer xenograft	(127)
	SFTI-FCQR (SFTI)	KLK4 inhibition	ovarian cancer cell line	(128)
	APPI-4M	KLK6 inhibition	breast cancer cell line	(129)
	DKFZ-251	KLK6 inhibition	pharynx carcinoma cell line	(130)
	Ac-GKAFRRL-12ADT	KLK2-targeting prodrug of thapsigargin	prostate cancer cell line	(131)
	L-377,202	KLK3-targeting prodrug of doxorubicin	prostate cancer	(132, 133)
	KCC-TGX	KLK3-targeting prodrug of TGX-221 (PI3Kβ) inhibitor	prostate cancer cell line	(134)
	^89^Zr-labeled 5A10 mAb	PET imaging	prostate cancer xenograft	(135)
	^111^In-DTPA-11B6 mAb	SPECT/CT imaging	prostate cancer xenograft	(136)
	KLK2/3/14_fABP	fluorescent imaging	prostate cancer cell line	(137)
Gzms	NOTA-GZP	PET imaging	colon cancer mouse model	(138, 139)
	^64^Cu-GRIP B	PET/CT imaging	colorectal cancer mouse model	(140)
	SK15.5	fluorescent imaging	breast cancer cell line	(141)
	qTJ71	fluorescent imaging	breast cancer cell line	(142)
	GzmB probe 1	chemiluminescent imaging	breast cancer mouse model	(143)
	GNR (nanoreporter)	NIR fluorescent imaging	colon carcinoma mouse model	(144)
	CyGbP_F_	NIR fluorescent imaging	breast cancer mouse model	(145)
	SPNP	NIR fluorescent and photoacoustic imaging	breast cancer mouse model	(146)

Given their high and selective expression on tumor-associated stromal cells, inhibition of serine proteases by active-site targeting small molecules has been extensively investigated in cancer treatment (30, 42, 102, 103, 115–121, 126–130). Antibody-based approaches that can inhibit the activity of serine proteases aim to achieve better selectivity. For example, a humanized version of monoclonal antibody F19, sibrotuzumab has been developed to target the cell surface bound FAP on tumor stromal fibroblasts and explored for its anti-tumor response (99). Protease biology in cancer offers opportunities for diagnostic profiling as well. Radiolabeled antibodies targeting FAP (106), uPA (122, 123), and KLK3 (135, 136) have been evaluated in single photon emission computed tomography (SPECT) and positron emission tomography (PET) imaging of primary and metastatic tumors. *In vivo* imaging tools exploit the cleavage activity of serine proteases. Incorporating the optimal peptidic sequence, substrate-based, activatable probes are designed either as quenched probes or as FRET probes that produce a fluorescent signal only after cleavage as a measure for protease activity (112, 113, 124, 142, 144–148). On the other hand, activity-based probes contain an electrophilic warhead that reacts with catalytic serine of the protease and a reporter group that generates a signal upon covalent interaction with the protease (137, 141, 149–152). In that case not only peptides but also non-peptidic small molecules can serve as a recognition motif targeting the protease active site (153–156).

Taking advantage of upregulation of specific serine protease activity in tumors, protease-activatable prodrug approach has been widely used in cancer therapy for selective delivery of drugs while minimizing toxicity to normal tissues. Chemotherapeutic compounds such as doxorubicin are conjugated to cleavable linkers sensitive to active serine proteases (104, 105, 131–134). For example, the FAP-cleavable GP linker and KLK3-cleavable SSKYQSL linker have been successfully used in prodrug approach. Antibody-drug conjugates that incorporate tumor-targeting antibodies into protease-activatable prodrug format further enhance tumor-specific activation. A new technology termed as Probody masks antibody binding using linkers cleaved by extracellular proteases. EGFR Probody PB1 only binds EGFR following cleavage by matriptase, uPA, or legumain (125).

While cancer immunotherapies have shown significant clinical outcomes, only a small subset of patients respond to the treatment, calling for reliable biomarkers and therapeutic strategies to maximize the benefits of the immunotherapy. Targeting the immunomodulatory, tumor stroma-associated serine proteases may provide a potential therapeutic option that complements and/or synergizes with the currently available immuno-oncology therapeutics. In cancer patients that high FAP expression in CAFs restricts T cell distribution and promotes immune checkpoint blockades (ICBs) resistance (41, 157, 158), treatment with FAP inhibitor could neutralize the immunosuppressive function of CAFs and reverse anti-PD1 drug resistance. Conditional depletion of FAP^+^CAFs could result in enhanced T cell infiltration and better response to anti-CTLA4 and anti-PD-L1 treatment. Injection of chimeric antigen receptor (CAR) T-cells constructed with anti-FAP mAb effectively depleted FAP^hi^ stromal cells and inhibited tumor growth. More importantly, FAP-specific CAR T cells augmented the antitumor responses of endogenous CD8^+^ T cells (159). The FAP-specific CAR T cell therapy lately advanced into a phase I clinical trial for treatment of patients with malignant pleural mesothelioma (NCT01722149).

Tumor-specific protease-activation can also be exploited to limit systemic exposure of immunotherapeutic agents and minimize unwanted immune toxicity and side effects. Recently, a dual variable domain (DVD) immunoglobulin of anti-CTLA4 antibody, that an outer domain (tumor targeting) is connected by serine protease cleavable linkers to an inner domain (CTLA4 targeting), has been developed (160). During systemic circulation, the CTLA4-binding site is shielded by the outer domain. Upon reaching the tumor, the outer domain is cleaved by membrane type-serine protease 1 (MT-SP1) present in the tumor microenvironment, leading to enhanced localization of anti-CTLA4 without causing significant treatment-associated toxicity. To deliver functionally active cytokines, that are found to be critical for establishing and maintaining the immune response to tumors, preferentially at the tumor site, the Frelinger group developed an activatable IL-2 fusion protein consisting of IL-2 joined to a specific IL-2 binder that blocks its function connected by a KLK3 cleavage sequence (161). This PACman (for *p*rotease *a*ctivated *c*ytokine) approach has been also applied to the generation of IL-12 fusion protein (162). Tumor-specific protease-activation strategy should be applicable to other TME proteases and immunomodulators allowing site-specific activation in immunotherapy.

A granule-associated serine protease GzmB has attracted significant attention as an early predictive biomarker for monitoring of immunotherapy responses. In particular, *in vivo* imaging approaches with activatable probes utilizing GzmB-cleavable IEPD or IEFD linker allow to directly report functional readouts of immune cell (e.g. CTLs and NK cells) infiltration, activation, and cytotoxicity in tumors after immunotherapy treatments (138–140, 143–146). Advanced protease-responsive technologies would enable more comprehensive exploration of proteolytic networks in cancer and provide the next generation of clinical modalities for cancer chemotherapy and immunotherapy.

## Conclusion

Understanding molecular mechanisms that orchestrate the complex and dynamic interplay between tumor cells and stromal microenvironment is important to provide insight into cancer biology. During malignant progression, several serine proteases appear to be key players at the tumor-stroma interface. These serine proteases function in multidirectional way by interacting not only with other proteases but also with important signaling pathways that involve activation or inactivation of cytokines, chemokines, growth factors, and kinases. The functions of serine proteases described in this review are not intended to be exhaustive but rather representative examples of recent discoveries. It is necessary to continue investigating the multifaceted roles proteases play within the tumor microenvironment in addition to their effects on the degradation of extracellular matrix to find more relevant diagnostic markers and therapeutic targets. Especially, in the context of anti-tumor immunity either naturally occurring or induced by immunotherapy, elucidating how these serine proteases on stromal cells are involved in modulating immune responses will help advance pharmaceutical strategies. Given the functional redundancy of proteases and cellular heterogeneity within the TME, advanced technologies that allow to dissect and manipulate specific serine protease with a spatiotemporal control will be highly required.

## Author Contributions

RT and EY made substantial contributions to conception and design of the review, contributed to the manuscript writing and revision, read and approved the submitted version.

## Funding

This work was supported by the Intramural Research Program of the NIH, National Cancer Institute, the Center for Cancer Research (ZIA BC011962).

## Conflict of Interest

The authors declare that the research was conducted in the absence of any commercial or financial relationships that could be construed as a potential conflict of interest.

## Publisher’s Note

All claims expressed in this article are solely those of the authors and do not necessarily represent those of their affiliated organizations, or those of the publisher, the editors and the reviewers. Any product that may be evaluated in this article, or claim that may be made by its manufacturer, is not guaranteed or endorsed by the publisher.
